# The Pathologic and Molecular Landscape of Esophageal Squamous Cell Carcinogenesis

**DOI:** 10.3390/cancers12082160

**Published:** 2020-08-04

**Authors:** Gianluca Businello, Paola Parente, Luca Mastracci, Gianmaria Pennelli, Giulia Traverso, Massimo Milione, Elena Bellan, Mauro Michelotto, Andromachi Kotsafti, Federica Grillo, Matteo Fassan

**Affiliations:** 1 Surgical Pathology Unit, Department of Medicine (DIMED), University of Padua, 35121 Padua, Italy; glc.businello@gmail.com (G.B.); gianmaria.pennelli@unipd.it (G.P.); bellan.elena@gmail.com (E.B.); mauro.michelotto@aopd.veneto.it (M.M.); 2 Pathology Unit, Fondazione IRCCS Ospedale Casa Sollievo della Sofferenza, 71013 San Giovanni Rotondo FG, Italy; paolaparente77@gmail.com; 3 Anatomic Pathology, Ospedale Policlinico San Martino IRCCS, 16132 Genova, Italy; mastracc@hotmail.com (L.M.); federica.grillo@unige.it (F.G.); 4 Anatomic Pathology, Department of Surgical Sciences and Integrated Diagnostics (DISC), University of Genova, 16132 Genova, Italy; massimo.milione@istitutotumori.mi.it; 5 Veneto Institute of Oncology IOV–IRCCS, 35128 Padua, Italy; me.giulia@gmail.com; 6 Laboratory of Advanced Translational Research, Veneto Institute of Oncology IOV–IRCCS, 35128 Padua, Italy; machikots@hotmail.com

**Keywords:** esophageal cancer, squamous cell carcinoma, biomarkers, molecular landscape, carcinogenesis

## Abstract

Esophageal squamous cell carcinoma represents the most common histotype of epithelial neoplasm occurring within esophageal mucosa worldwide. Despite the comprehensive molecular characterization of this entity, to date no significant targeted therapy has been introduced into clinical practice. In this review, we describe the molecular landscape of esophageal squamous cell carcinoma based on the most recent literature. Moreover, we focus on other rare variants and on the relationship with head and neck squamous cell carcinomas.

## 1. Introduction

Esophageal cancer (EC) is the sixth leading cause of cancer-related death worldwide, with 442,000 new cases and 440,000 deaths in 2013 and with 5-year overall survival rate ranging from 15 to 25% [1,2]. Esophageal squamous cell carcinoma (ESCC) is the most common histotype, representing nearly 90% of all ECs, with a global incidence of 5.2 per 100,000 [3]. Globally, ESCC is more common in men (69%) than women (31%) [4].

There are marked geographic differences in the incidence of ESCC. Areas with the highest incidence rates (13.6 per 100,000 in men and 4.3 in women) are the so-called “cancer belt” (i.e., Northern Iran, Central Asia and China) and the Indian Ocean coast of Africa [3]. High incidence rates (5.9–6.4 per 100,000 in men and 3.6–4.0 in women) are also found in Sub-Saharan Africa and Central Asia. Intermediate rates (4.3–4.7 per 100,000 in men and 0.6–1.3 in women) are observed in Eastern Europe and the Caribbean, Central and South America. In contrast, Northern, Western and Southern Europe, North America, Oceania, North Africa and Western Asia are characterized by lower incidence rates (less than 3.8 per 100,000 in men and 1.7 in women) [3]. Interestingly, in the last 40 years the incidence of ESCC in Western countries has been decreasing, whereas the incidence of esophageal adenocarcinoma (EAC) has increased [4].

Both environmental and inherited risk factors for ESCC onset are known, with different impact in distinct geographic regions.

Consuming tobacco products is a well-known risk factor, higher in economically developed countries than in developing countries (relative risk in current smokers of 3–9 vs 1.5) [4]. Likewise, drinking alcoholic beverages is also associated with an increased risk of 1.6–5.6-fold in Asian countries, 3-fold in Africa and South America, 6-fold in Europe and 9-fold in North America [4]. Notably, tobacco and alcohol have a synergistic effect, with a multiplicative risk product of both [5].

In addition, polycyclic aromatic hydrocarbons (PAHs) are strongly associated with an increased risk of ESCC: Iranian, Brazilian and Chinese populations are highly exposed to PAHs coming from food or beverages [4,6]. Betel quid (consumed typically in South Asia) [7] and the consumption of hot beverages (which is in the cultural background of many countries, such as in South America and in Asia) are also associated with an increased risk of ESCC [8]. Finally acquired risk factors for ESCC include a low socioeconomic status, poor oral hygiene and some medical conditions such as achalasia [4,9].

Several evidences show that many hereditary factors are involved in the etiology of ESCC. High risk of ESCC (up to 95% by age 65) is described in *RHBDF2*-associated tylosis, an autosomal dominant disorder characterized by palmoplantar keratoderma [10], in Plummer–Vinson syndrome [4] and in Fanconi anemia [11]. Moreover, individuals carrying specific variants of *ALDH2*, the aldehyde dehydrogenase 2 family genes, have a higher risk of ESCC if alcohol assumption is added (43-fold in moderate drinkers and 73-fold in heavy drinkers); these genetic alterations are typically found in people from East Asia [4,12]. Several key single nucleotide polymorphisms (SNPs) of *PLCE1* have been associated with an higher risk of ESCC: of note, *PLCE1* encodes for the 1-phosphatidylinositol 4,5-bisphosphate phosphodiesterase epsilon-1, a protein which appears to have a role in modulating carcinogenetic effects [4].

Golyan and colleagues [13] suggest that *CDK11A*, *PHLDA1*, *CDKN2AIP*, *MAML3*, *ARID1A* and *JMJD6* are candidate genes involved in ESCC genetic predisposition in patients with familial ESCC.

Finally, a SNP of *TP53*—the most commonly mutated gene in ESCC (vide infra)—has been associated with a higher risk of this cancer [14].

## 2. Squamous Preneoplastic Lesions and Squamous Carcinogenesis within the Esophageal Mucosa

**Figure 1:**
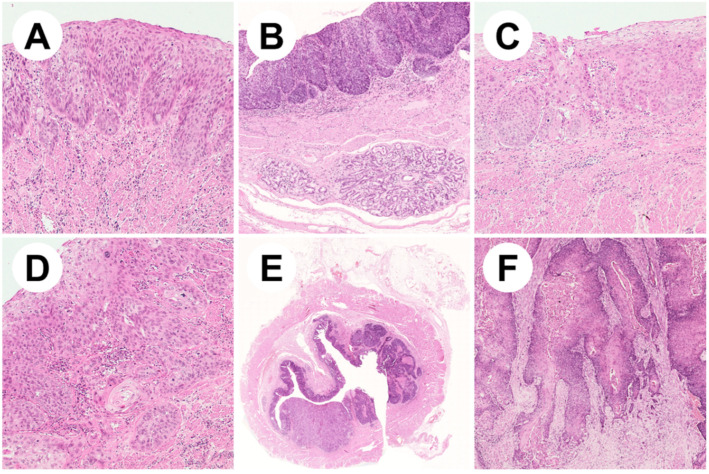
Representative pictures of esophageal squamous neoplastic lesions. (**A**) Low-grade squamous cell dysplasia showing involvement of the lower half of the epithelium with mild cytological atypia (ematoxylin/eosin, 10×); (**B**) high-grade dysplastic epithelium covering esophageal submucosal glands (ematoxylin/eosin, 5×); (**C**) high-grade dysplasia with full-thickness wall involvement and severe cytological atypia (ematoxylin/eosin, 10×); (**D**) early invasive squamous cell carcinoma (ematoxylin/eosin, 20×); (**E**) low magnification appearance of a squamous cell carcinoma of the middle esophageal wall (ematoxylin/eosin, 1×); (**F**) well-differentiated keratinizing squamous cell carcinoma (ematoxylin/eosin, 10×).

The progression from normal squamous esophageal epithelium to ESCC is the result of a multistep process that ideally starts with basal cell hyperplasia and, through dysplasia, culminates in invasive carcinoma (Figure 1) [15]. Each step is histologically recognizable following this process with screening programs in high risk patients to achieve an early diagnosis with curative treatment intent of ESCC [16]. Squamous dysplasia (SD) (i.e., an unequivocal neoplastic alteration of the esophageal squamous epithelium without invasion of the basal membrane) is the only validated precursor lesion of ESCC [16,17].

Squamous dysplasia (SD) is histologically defined by both cytological (i.e., nuclear enlargement, pleomorphism, hyperchromasia, loss of polarity, overlapping) and architectural atypia (i.e., abnormal epithelial maturation) [15]. SD is graded using a two-tier system (low- vs high-grade), according to 2019 WHO Classification of Tumors of Digestive System: dysplasia limited to the lower half of the epithelium with mild cytological atypia is classified as ‘low grade’, while ‘high grade dysplasia’ is diagnosed if dysplasia extends to the upper half of the epithelium or if severe cytologic atypia is documented [17]. Carcinoma in situ (i.e., dysplastic squamous cells involving the full thickness of the epithelium without invasion of basal membrane) is included in the high grade dysplasia category [15,17].

The identification of molecular alterations leading to tumorigenesis in basal cell hyperplasia has been challenging. Liu and colleagues [18] found several ESCC-associated mutations in basal cell hyperplasia, such as in the *TP53*, *NOTCH1*, *CDKN2A*, *EP300* and *MLL2* genes. Both low- and high-grade dysplasia are characterized by a higher number of genomic and epigenomic alterations. Large-scale chromosomal deletions at 9p21.3 (*CDKN2A*) and 2q35 and amplifications at 11q13.3 (*CCND1*), 5p15.33, 8q24, 2q31.2 (*NFE2L2*), 8p11.23, 7q22.1 and 3q27 (*SOX2*) have been described in dysplastic esophageal tissues and are thought to be early events in ESCC carcinogenesis [18]. Moreover, loss of heterozygosity (LOH) of *TP53* has been reported in 30% of esophageal dysplastic tissues and LOH of *CDKN2A* (15%), *NOTCH* family genes (19%), *RB1* (11%) and *YAP1* have also been described [18]. Furthermore, mutations of *TP53* (71% of esophageal dysplastic samples), *NOTCH1*, *CDKN2A*, *PIK3CA*, *EP300* and *MLL2* have been reported [18]. Alterations in the expression of those genes are commonly found in ESCC (vide infra). Finally, promoter hypermethylation of tumor suppressor genes *CDKN2A*, *CLDN3*, *MT1G*, *DAPK*, *MGMT*, *MLH1*, *RARβ2*, *HIN1*, *TFPI-2*, *DACH1* and *SOX17* have been documented [19,20,21].

Specifically, the transition from normal squamous mucosa to dysplasia starts with the expansion in normal mucosa of many independent precancerous clones, generated by a process of positive selection and sustained by the acquisition of driver mutations [22,23]. This process has been described by Yokoyama and colleagues [24]. According to their results, clonal expansion in normal esophageal epithelium is a consequence of normal aging but can be accelerated by alcohol and tobacco consumption. Briefly, clones start to develop in infancy and increase their number and size over the years, eventually remodeling all the esophageal epithelium, with about 9000–15,000 clones per esophagus [24]. The mutational analysis revealed significant differences in the frequency of mutation of many genes between normal and dysplastic epithelium. *TP53*, *NFE2L2*, *CDKN2A* and *FBXW7* were more frequently mutated in ESCC or dysplastic samples, while *NOTCH* family genes, *PPM1D*, *ZFP36 L2*, *FAT1*, *CHEK2* and *PAX9* were more frequently mutated in normal epithelium. In particular, *NOTCH1* was the most frequently mutated gene in normal esophageal mucosa (66.2% of cases), contrasting to the relatively low frequency of mutation detected in ESCC (15.0%). These differences support the hypothesis of a mechanism of positive selection occurring in normal versus neoplastic epithelium [24]. Finally, uniparental disomy and LOH of 9q, gain of 3q and LOH of 17p have been described in normal esophageal mucosa [24].

## 3. Molecular Landscape of ESCC

ESCC genome holds a wide variety of genetic alteration types ranging from single point mutations to chromosomal structure variations, some of which have a pivotal role in carcinogenesis. Moreover, a growing body of evidence is defining epigenetic dysregulation as a main actor in ESCC development.

### 3.1. Genetic Landscape of ESCC

#### 3.1.1. Gene Mutations

Gene mutations, leading to loss or alteration of gene function, play an important role in ESCC carcinogenesis. The average mutation rate has been estimated to be 3.9 coding mutations/Mb in whole genome sequencing and 2.4 non-silent mutation/Mb in whole exome sequencing, with a median of 97 non-silent mutations per tumor [25,26]. Many genes are mutated in ESCC and, until now, at least 18 significant mutated genes (SMGs, i.e., genes that show a significantly higher mutational rate than the background mutation rate) have been identified and well characterized. The better understood SMGs in ESCC can be categorized according to their functions in: (i) genes involved in cell cycle regulation (*TP53*, *CDKN2A*, *RB1*, *CREBBP*), (ii) genes involved in the PI3K/AKT/mTOR pathway (*PIK3CA*, *PTEN*), (iii) genes involved in the NOTCH signaling pathway (*NOTCH1*, *NOTCH3*, *FBXW7*), iv) genes involved in cell adhesion (*AJUBA*, *FAT1*), v) genes involved in chromatin remodeling (*MLL2*, *KDM6A*, *EP300*, *BAP1*), (vi) genes involved in the NRF2 pathway (*NFE2L2*, *CUL3*) and vii) genes involved in epidermal differentiation (*ZNF750*) [25,26,27,28,29,30,31]. A comprehensive view of all SMGs in ESCC is reported in Table 1.

##### Mutational Signature

Analysis of the mutational spectrum of ESCC established that C:G>T:A transition are the predominant type, followed by C:G>A:T and C:G>G:C transversions [26,33]. In order to better understand the mutational processes involved in ESCC carcinogenesis, some Authors unveiled different mutational signatures using a mathematical procedure known as non-negative matrix-factorization method: until now, at least six signatures (Signatures 1, 2, 4, 5, 13 and 16, named according to the Catalogue Of Somatic Mutations In Cancer (COSMIC) nomenclature) have been described in ESCC. Signature 1 is defined by an enrichment of C>T mutations in XpCpG trinucleotides, a well-recognized mutational process related to spontaneous deamination of 5-methyl-cytosine [25,26]. Signature 2 and Signature 13 are characterized by C>G and C>T/C>A mutations in TpCpX trinucleotides, respectively and are associated with mutations of the APOBEC (Apolipoprotein B mRNA Editing Catalytic Polypeptide-like) family of cytidine deaminases, which have important roles in immunologic processes due to their deaminase activity [25,26,28,34]. Signature 4 is probably due to tobacco carcinogens and is characterized by an augmented rate of C>A mutations [26,27,35,36]. Signature 16 is defined by T>C mutations in ApTpX trinucleotide and has been associated with alcohol consumption [28,35].

##### Genes Involved in Cell Cycle Regulation

Furthermore, known as “the guardian of the genome”, p53 is a well-studied protein which plays a crucial role in cell cycle regulation, DNA repair and cell apoptosis. *TP53* is frequently mutated in many cancers and is the most frequently mutated gene in ESCC (85–93% of cases) [25,26,32,33]. *CDKN2A* is another well-known tumor suppressor gene inhibiting cell cycle progression through its interaction with both the p53 and the Rb pathways, and it is mutated in 4.9 to 20% of ESCCs [26,32,33,37]. The cell cycle regulator gene *RB1* is mutated in 4.2 to 9% of ESCC [26,32,33]. *CREBBP*, mutated in 5.9 to 7.6% of ESCC, plays a role in transactivation of p53 [32,33,35,38].

##### Genes Involved in PI3K/AKT/mTOR Pathway

The PI3K/AKT/mTOR pathway is a well-understood intracellular signaling pathway with an important role in cell cycle progression. Genes involved in this pathway, specifically the SMGs *PIK3CA* (10.8–17%) and *PTEN* (3%), are reported to be mutated in 29% of ESCCs [25,26]. *PIK3CA* encodes for PI3K, an intercellular mediator of cell survival signals and functions as an oncogene leading to AKT activation and, consequently, mTOR phosphorylation. *PTEN* acts as a tumor suppressor gene inhibiting AKT dephosphorylation [26]. Hotspot mutations in the p110a domain (p.N345K, p.C420R, p.E545K, p.E542K) and in C-terminal portion (p.H1047R, p.H1047L) encoding portions of the *PIK3CA* gene have been detected, which are reported to induce a gain of function in oncogenicity [26]. Another study found that hotspot mutations c.1624G>A[p.E542K] and c.1633G>A [p.E545K] on *PIK3CA* were significantly enriched in ESCCs with Signatures 2 and 13, suggesting that APOBEC activity is a driver of *PIK3CA* mutagenesis in ESCC [25].

##### Genes Involved in the NOTCH Signaling Pathway

Many biologic functions are regulated by the NOTCH signaling pathway and its role in many malignant tumors—the most common T-lymphoblastic leukemia—is complex and not completely understood [39]. Alterations in NOTCH signaling pathways have been reported in up to 33.4% of ESCCs; in particular, the SMGs *NOTCH1*, *NOTCH3* and *FBXW7* are mutated in 16%, 6% and 5% of ESCC, respectively [26,32,33]. Interestingly, *NOTCH1* mutations in ESCC are clustered within epidermal growth factor-like repeats 11–12, involved in ligand binding. The mutational hot-spot region is different from the HD (heterodimerization) and PEST (proline, glutamic acid, serine, and threonine-rich) domain harboring the oncogenic mutation in T-lymphoblastic leukemia, but it is similar to the region harboring putative tumor suppressive mutations in other types of squamous cell carcinoma [33].

##### Genes Involved in Cell Adhesion

*AJUBA* encodes a LIM (Lin-11, Isl1 and Mec-3)-domain protein which is involved in several cellular processes, such as cell adhesion, cytoskeletal organization, cell fate determination, mitotic commitment, inhibitions of ATR (ataxia telangiectasia and Rad3-related protein)-dependent DNA-damage response and has been proposed to be a major component of the miRNA-mediated gene-silencing machinery [40,41]. Its role in malignancies is poorly understood as it has been reported to act both as an oncogene and a tumor suppressor gene in different types of cancers [25,42]. *AJUBA* is mutated in 3.9 to 7% of ESCCs. Two stop-gain mutations (c.985G>T [p.E329*] and c.1057C>T [p.Q353*]) and two frameshift indels (c.790_791insT [p.V264fs*] and c.152delG [p.G51fs*]) in *AJUBA* gene have been identified [25]. These mutations result in protein products that lack the LIM domain, indicating that they are loss-of-function mutations: since mutated *AJUBA* seems to promote ESCC carcinogenesis, these data suggest that *AJUBA* has a tumor suppressive role in ESCC [25].

*FAT1*, mutated in 10 to 15% of ESCC, encodes for a cadherin-like protein and is reported to regulate cell adhesion by controlling actin polymerization [25,26,33].

##### Genes Involved in Chromatin Remodeling

Histone-modifying enzymes control chromatin structure and regulate gene expression: mutations of these enzymes play an important role in carcinogenesis [43]. Four SMGs in ESCC involved in chromatin remodeling have been discovered: *MML2* (mutated in 11 to 19% of ESCC), *KDM6A* (3–7%), *EP300* (8–13%) and *BAP1* (less than 5%) [25,26,28,32,33].

##### Genes Involved in the NRF2 Signaling Pathway

The NRF2 signaling pathway is composed by *NF2EL2*, *KEAP1* and *CUL3.* Mutations of these genes have been observed in many squamous cell carcinomas, ESCC included [44]. *NFE2L2* encodes a transcription factor involved in cellular response to oxidative stress while *KEAP1*/*CUL3* (Kelch-like ECH-associated protein 1/Cullin3)-mediated ubiquitination degrades NFE2L2 (nuclear factor erythroid-derived 2-like 2) protein in normal conditions, thus making the cell more vulnerable to oxidative damage [45]. Furthermore, *CUL3* mutations or deletion have been associated with upregulation of beta-catenin with concordant changes in Wnt-beta catenin downstream factors such as c-MYC, cyclin D1 and p27 [27]. Mutations in NRF2 signaling pathway have been described in 24% of ESCC, in particular the SMGs *NFE2L2* and *CUL3* are mutated in 9.6 to 16.7% and 2.9% of cases, respectively [26,32,33].

##### ZNF750

*ZNF750* encodes for an epidermal differentiation regulator and has been proposed to work as a tumor suppressor gene in ESCC [25]. *ZNF750* is reported to be mutated in 3.9 to 16.7% of ESCC [25,26,33].

##### Other Mutated Genes

Many other mutated genes have been described in ESCC, such as *DCDC1*, *FAM135B*, *ADAM29*, *FAT2*, *TGFBR2*, *NAV3*, *TENM3*, *TET2*, *PTCH1*, *USP8*, *RIPK4*, *PBRM1* and *VANGL1*. Despite some studies having characterized them well, their role in ESCC is still poorly understood [26,27,28,30,31,33,46]. Of note, *TENM3* mutation has been associated with poorer outcome [28].

#### 3.1.2. Structural Variants (SVs)

Chromosomal SVs are common events in ESCC, with an average number of 73 to 168 SVs per tumor [35,47]. Chromosomal translocations and deletions are the most common SVs in ESCC, being detected in 27 to 42% and 12 to 35% of cases, respectively. Nevertheless, inversions, duplications and insertions have also been described [35,47]. Non homologous end joining, and microhomology-mediated end joining are considered the dominant mechanisms leading to translocations and deletions [47]. Noteworthy, the mutational processes of chromothripsis, breakage fusion bridge and kataegis also have a role in ESCC development [35,47]. Copy number variations (CNVs) at chromosomal arm level are found in 98% of ESCC: in particular loss of 3p, 4p, 4q, 9p, 13q, 18q, 19p and gain of 3q,5p, 7p, 8q, 12p, 16p, 20p, 20q and 22q have been frequently described [35,48,49].

##### Gene Amplifications

Gene amplification is one of the leading causes of proto–oncogene activations, playing a crucial role in carcinogenesis. In ESCC many gene amplifications have been described, some of which have clinical relevance.

*CCND1* (11q13.3) amplification is common in many tumors and has been associated with lymph node metastasis in ESCC and poorer clinical outcome [31,47,50]. *EGFR* (7p11.2) has an amplification rate of 7% to 15% and is associated with poor prognosis [31,51,52], as *FGFR1* (8p11.23) amplification [31,53,54]. *SOX2* (3q26.33) amplification has been found in 15% of ESCC and it has been postulated that its downregulation may inhibit ESCC carcinogenesis and improve the efficacy of chemotherapy [25,29,55]. *TP63* gene encodes a squamous transcription factor; amplifications of *SOX2* or *TP63* were reported in 48% of ESCCs [29]. Other amplified genes involved in ESCC are *MYC* (8q24.21), *KRAS* (12p12.1), *MDM2* (12q15), *PIK3CA* (3q26.32), *YAP1* (11q22.1), *CBX4* and *CBX8* (17q25.3) [25,29,31,56].

##### Gene Deletions

*CDKN2A*/*2B* (9q21.3) is involved in cell cycle regulation and is deleted in 36% to 48% of ESCCs [33,35]. *FBXW7* is a well-established tumor suppressor gene and its product is involved in cell cycle regulation via degradation of many oncogenic proteins such as c-myc. Deletion of *FBXW7* (4q31.3) has been described in 6% to 45% of ESCC and seems to be associated with a worse prognosis [35,57]. Deletions of *TP53* (17p13.1) have been detected in 55% of ESCCs, correlating with the grade of differentiation and lymph node metastasis [58]. Other genes deleted in ESCC are *LRP1B* (2q22.1–22.2), *TNFRSF10A* (8p21.3), *PDE4D* (5q12.1), *PTPRD* (9q24.1), *FHIT* (3q14.2), *RB1* (13q14.2), *PTEN* (10q23.21), *PIK3R1* (5q13.1), *FAT1* (4q35.2), *ZNF750* (17q25.3), *CUL1* (7q36.1), *CUL5* (11q22.3) and *VGLL4*/*ATG7* (3p25.2) [29,31,33,35].

### 3.2. Epigenetic Landscape of ESCC

The study of epigenetic alterations (i.e., stable alterations in gene expression without underlying genic sequence modifications) is one of the most intriguing and expanding fields, important specifically in cancer research [59]. Increasing evidence suggest that epigenetic alterations plays an important role in the development of many malignancies, including ESCC [59]. Furthermore, RNA editing process seems to be dysregulated in ESCC, since overexpression of *ADAR1*, a RNA-specific adenosine deaminase, has been reported [60]. Despite epigenomic studies being relatively recent, many factors involved in ESCC carcinogenesis have been identified, particularly in the fields of DNA methylation and microRNA (miRNA) regulation.

#### 3.2.1. DNA Methylation

A growing body of evidence is the basis for better understanding the mechanisms and the significance of DNA methylation in ESCC carcinogenesis. The DNA methylation profile of ESCC genome is characterized, similarly to other human malignancies, by a widespread hypomethylation and site-specific CpG island promoter hypermethylation [59]. Interestingly, DNA methylation can regulate gene expression directly via gene promoter methylation and indirectly through the promoter methylation of other regulator mechanisms such as miRNA [61].

Site-specific CpG island promoter hypermethylation, silencing tumor suppressor genes, contributes to ESCC carcinogenesis. Promoter hypermethylation of different genes has been described in ESCC and some of them seem to have clinical relevance. The promoter of *APC*, a well know tumor suppressor gene, is hypermethylated in 27% to 46% of ESCCs [62,63,64]. *APC* hypermethylation status has controversial clinical relations: it has been described to be associated with a reduced survival time [62], and with a lower number of nodal metastases and better prognosis [63]. Hypermethylation of *CDH1*, the gene encoding for E-cadherin, is reported in 14% to 61% of ESCCs and has been associated with poor/lower recurrence-free survival in early stage ESCCs [65,66,67,68]. Methylation-induced inactivation of *CDKN2A* is reported in 19% to 88% of ESCCs and has been associated with metastatic disease [59,65,67,69]. *FHIT* (3p14.2) encompasses FRA3B and encodes for a tumor suppressor gene: its inactivation is reported in many cancers, even if the exact mechanism is as yet not completely understood [70]. Hypermethylation of *FHIT* promoter is reported in 14% to 85% of ESCC and has been associated with aggressive forms and poor prognosis in early ESCC [59,71,72] and with exposure to tobacco smoke [72]. *MGMT*, encoding for a DNA repair enzyme, is hypermethylated in 27% to 72% of ESCCs and has been related to lymph node metastasis [59,73], but in esophageal carcinomas an enhanced response to temozolomide treatment has been reported [74]. Lu and colleagues [75] suggest that hypermethylation of *CCD8* and *FBXO17* is significantly associated with a poorer prognosis, while hypermethylation of *ABCD1* correlates with a better one. Aberrant methylation of a gene coding for a F-box protein, *FBOX32*, have been associated with poorer 5-year survival [76]. Wang and colleagues [77], analyzing samples from Chinese Han patients, demonstrated that *ADHFE1*, *EOMES*, *SALL* and *TFPI2* are hypermethylated in ESCCs, and hypomethylated in the corresponding non-neoplastic tissues. Hypermethylation of *TFF1*, a mucosal protective factor, seems to be an early event in ESCC development and, intriguingly, could be used as a biomarker for early ESCC detection [78]. Methylation of *IGFBPL1* have also been proposed as an early detection marker and a predictive marker for PI3K-targeted therapy [79]. Finally, hypermethylation of many other genes, such as *MSH3*, *HIN-1*, *DAB2*, *RUNX3*, *RASSF1A*, *RASSF10*, *SOX17*, *DACT2*, *ZN331*, *RARB*, *MLH1* and *MSH2*, has been reported, but their clinical impact is unclear [21,59].

Global hypomethylation status contributes to carcinogenesis in many different malignancies by activating some proto–oncogenes, leading to deletions and translocations, promoting mitotic recombination, chromosomal rearrangements and, in general, resulting in genomic instability [59,80]. Nevertheless, the role of hypomethylation in ESCC is more poorly understood compared to hypermethylation. Some studies have investigated the methylation level of the long interspersed nuclear element-1 (LINE-1), which is considered as good indicator of the global methylation status [81,82,83,84]. LINE-1 hypomethylation in ESCC has been associated with lymph node metastasis, lymphovascular invasion, increased frequency of *TP53* mutations, higher CDK6 protein expression levels and a shorter overall survival [82,83,84]. However, more studies are needed to assess the importance of those results, since LINE-1 hypomethylation itself does not elucidate the impact of alterations of methylation in functional genomic domains [85].

#### 3.2.2. MicroRNA

MiRNAs are a family of 21 to 25-nucleotide non-coding RNAs that regulate gene expression in post transcriptional phases, regulating different cellular processes [86]. MiRNAs are a promising field of interest, since their study could lead to a better understanding of tumorigenesis and help in finding new diagnostic or therapeutic biomarkers. Alterations in miRNA expression are involved in different malignancies, affecting cellular processes of proliferation, motility, invasion and apoptosis [87,88]. Downregulation of tumor-suppressive miRNAs cause the overexpression of oncogenes, while overexpression of onco-miRNAs inhibits different tumor suppressor genes. These mechanisms contribute to the acquisition of a malignant phenotype in neoplastic cells [88]. Next generation sequencing-based profiling of miRNA in ESCC samples reports at least 78 dysregulated miRNAs [89]. The most frequent miRNAs involved in ESCC are reported in Table 2 [88,89,90]. Some miRNAs, moreover, may have a role in chemosensitivity of ESCC. Overexpression of miR-200c, miR-96, miR-141 and miR-27 has been associated with resistance to platinum-based chemotherapy [91,92,93,94] while combined downregulation of miR-133a and miR-133b increase the sensitivity to paclitaxel-based chemotherapy [95].

As previously asserted, since the prognosis of ESCC is strongly related to disease stage, there is interest in the development of sensitive and low-cost screening to detect patients at early stages. Given its resistance in biologic fluids, circulating miRNA tests could be promising, non-invasive methods to achieve this goal [96]. In a meta-analysis, Zhang and colleagues [96] report an overexpression on miR-21 and miR-223 and a reduced expression of miR-375 and miR-100 in ESCC patients’ plasma compared with controls and suggest miRNA can be used as biomarkers for ESCC diagnosis. Sudo and colleagues [97] propose a panel of six miRNA as a serologic test to diagnose ESCC at early stage: in their results, serologic levels of miR-8073, miR-3196 and miR-744-5p were higher, while serological levels of miR-6820-5p, miR-6794-5p and miR-6799-5p were lower in ESCC patients than in controls. Finally, levels of circulating miR-1233, miR-6885-5p, miR-4698 and miR-128-2-5p have predictive significance, being associated with response to nivolumab [98].

### 3.3. Genetic Comparison between EAC and ESCC

The two main histotypes of EC, ESCC and EAC, are different in their molecular landscapes. According to recent studies, EAC has more resemblance with the CIN subtype of gastric cancer than with ESCC [29,99]. The main molecular alterations in EAC are briefly described below. Well characterized SMGs in EAC are *TP53*, *CDKN2A*, *ARID1A*, *SMAD4* and *ERBB2* [29,100]. Despite *TP53* and *CDKN2A* are frequently mutated as in ESCC, *ARID1A*, *SMAD4* and *ERBB2* mutations occur preeminently in EAC [29,99]. Moreover, inactivating mutations of *NOTCH1* have been described in ESCC, but not in EAC [101]. Gene expression analysis unveiled significant differences in the expression of molecular pathways between EAC and ESCC: *CDH1* signaling together with E-cadherin regulator *ARF6* and *FOXA* pathways are upregulated in EAC, while ESCC is associated with the upregulation of Wnt, syndecan and p63 pathways [29]. Substantial differences in SVs have also been reported. Amplifications of *VEGFA* (6p21.1), *ERBB2* (17p12), *GATA6* (18q11.2) and *CCNE1* (19q12) are significantly more frequent in EAC than in ESCC [29]. On the other hand, amplifications of *FGF3*, *FGF4*, *FGF19*, and *CCND1* (colocalized on 11q13) and *FGFR1* (8p11.23) have been more frequently described in EAC [99]. Finally, deletion of *SMAD4* (18q21.2) is recurrent in EAC, but not in ESCC [29]. All those differences suggest different therapeutic approaches to EAC and ESCC and recommend extreme caution when a clinical trial in mixed EAC and ESCC populations is performed.

## 4. Rare Histopathologic Variants of Squamous Cell Carcinoma and Their Molecular Background

**Figure 2:**
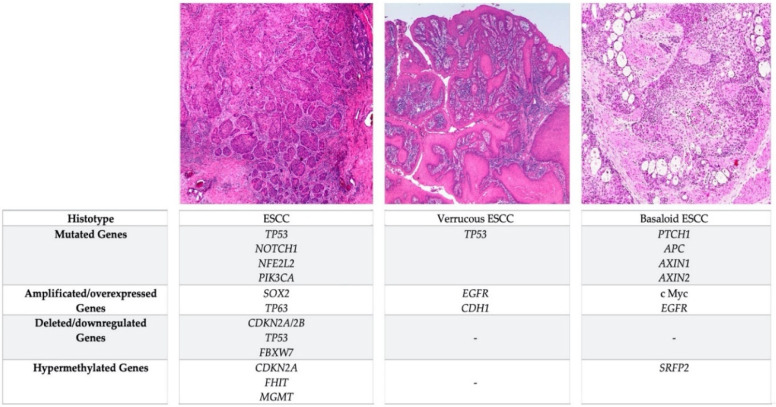
Comparison of the most frequent genetic and epigenetic alterations occurring in ESCC and in the verrucous and basaloid variants.

Rare ESCC variants (i.e., verrucous esophageal carcinoma, spindle cell squamous cell carcinoma and basaloid squamous cell carcinoma) are histologically well characterized and their clinical impact is relatively well known [17]; conversely, their carcinogenetic sequence remains unclear, since the molecular alterations involved in their development are not satisfactorily understood [102,103,104]. Verrucous esophageal carcinoma and basaloid esophageal squamous cell carcinoma are the two variants better characterized in their molecular alterations are described below (Figure 2).

### 4.1. Verrucous Esophageal Carcinoma

Verrucous esophageal carcinoma (VEC) is a rare variant of ESCC with puzzling etiological, clinical and molecular features. VEC is associated with the same risk factors of ESCC and typically affects the middle-aged population, with a male to female ratio of 2:1 [105,106]. The role of human papilloma virus (HPV) in VEC carcinogenesis is controversial, but the most recent study suggests that HPV is not involved in VEC [102]. Histologically, VEC is an exophytic and well differentiated lesion, with a lymphocyte rich pushing border and heavily hyperparakeratinized epithelium or an irregular clefted surface with keratin plugging extending deeply into the clefts [106,107]. The molecular alterations are neither extensively studied nor understood yet. Cappellesso and colleagues [102] provide a comprehensive investigation of the molecular alterations and immunohistochemical profile occurring in 9 cases of VEC. Low EGFR immunohistochemical expression was found in 5 patients and in one metastatic case, while a moderate expression was found in 2 cases. These data seem to be in accordance with the worse prognosis of *EGFR* overexpression in classic ESCC [51]. On the other hand, cyclin D1 expression was low in 3/9 tumors included the metastatic one, in apparent discordance with the prognostic significance of cyclin D1 overexpression in classic ESCC. E-cadherin expression was high in 6/7 tumors, moderate in two and low in the metastatic one. Overexpression of p16 was found in 2 tumors, while significant p53 nuclear immunostaining in more than 75% of neoplastic cells has been found in 4 cases. Interestingly, *TP53* mutation has been identified only in the metastatic case. The *TP53*-mutated VEC harbored a heterozygous point mutation in position c.738G >A of exon 7 of *TP53*, resulting in the mutant variant p.M246I of p53. These data suggest that *TP53* missense point mutations, *EGFR* overexpression and E-cadherin downregulation may have a role in VEC progression and metastasis [102].

### 4.2. Basaloid ESCC

Basaloid ESCC is a rare and poorly defined variant of ESCC, accounting for 2% of all esophageal malignancies [108]. Histologically, basaloid ESCC is characterized by solid nests of cells with scant cytoplasm and hyperchromatic nuclei with comedo-type necrosis, lobular or trabecular architecture, cribriform/pseudo glandular pattern [109]. The molecular pathogenesis is still unclear, but, interestingly, there are some differential characteristics with classical ESCC [103]. Expression of p53 is reported in at least half of the cases and overexpression of p53 has been suggested to be less frequent compared to classical ESCC [110]. In addition, *CDKN2A* product (i.e., p16) expression is less common in basaloid histotype [110]. Conversely, bcl-2 expression and c-myc amplification are more common in basaloid ESCC than in conventional ESCC [111]. Mutually exclusive *EGFR* mutations or amplifications have been reported [109]. Furthermore, activation of Wnt signaling pathway is common [112] and unrelated to mutations in *CTNNB1*; mutations in *APC*, *AXIN1* or *AXIN2* genes and by hypermethylation of the *SRFP2* gene promoter seem to be involved in this process [112]. Finally, mutations in *PTCH1* have been reported in nearly 53% of basaloid ESCC [113]. Alterations of *PTCH1* lead to constitutive activation of the hedgehog signaling pathway and germinal mutation of *PTCH1* are linked to Gorlin syndrome (i.e., nevoid basal cell carcinoma syndrome) [113,114].

## 5. Relationship with Head and Neck Tumors and Synergic Molecular Alterations

Head and neck squamous cell carcinoma (HNSCC) is a basket category including a group of squamous neoplasms arising from the mucosal surface of nasal cavities, paranasal sinuses, oral cavity, nasopharynx, oropharynx, hypopharynx and larynx. HNSCC represents 90% of malignancies arising in the head and neck region and is the sixth most common malignancy worldwide [115]. Other than the obvious histopathologic similarity, HNSCC shares many features with ESCC and 2.7 to 12.5% of patients with HNSCC develop synchronous ESCC [116,117,118,119]. The molecular mechanisms underlining second primary malignancies’ development in patients with HNSCC is not completely clear, but the concept of “field cancerization” helps to explain this event: in the upper aerodigestive mucosa, the constant exposure to carcinogenic factors leads to the development of multiple areas (i.e., “fields”) harboring genetic aberrations which are prone to malignant transformation [120]. Alcohol and tobacco products are major carcinogenic agents for both ESCC and HNSCC, being associated with 70–80% of new diagnosed HNSCCs [121]. Common risk factors for ESCC and HNSCC are also poor oral hygiene and betel quid consumption [122]. HPV, in particular HPV-16 subtype, has an important role in HNSCC carcinogenesis, but not in ESCC: 20 to 25% of NHSCC are associated with HPV which is a highly favorable prognostic indicator [123,124].

Many molecular pathways altered in ESCC are commonly dysregulated in HNSCC as well [122]. Both ESCC and HNSCC share alterations in genes involved in cell cycle regulation (e.g., *TP53*, *RB1*, *CDKN2A*, *CCND1* and the PI3K/AKT/mTOR pathway), NOTCH pathway (e.g., *NOTCH1*) and cellular adhesion (e.g., *FAT1*). Not surprisingly, these common features with ESCC are more marked in HPV-negative rather than in HPV-positive HNSCCs [122,125,126].

### 5.1. Structural and Copy Number Variants

Common SVs in ESCC and HNSCC are loss of 3p and gains of 3q, 5p and 8q [126]. Specifically, HPV-negative HNSCC are characterized by deletions of *CDKN2A*, *FAT1*, *NOTCH1*, *SMAD4* and amplification of *EGFR*, *ERBB2*, *CCND1* and *FGFR1* [126]. In particular, *EGFR* is overexpressed in 80 to 90% of HNSCCs and is related with poor prognosis as in ESCC [127]. Amplification of 3q 26–28 have been described in both HPV positive and negative HNSCCs [126]. This region harbors the squamous lineage transcription factors *TP63* and *SOX2*, as well as the oncogene *PIK3CA*, suggesting its pivotal role in squamous cellular differentiation. HPV-positive HNSCCs have been associated with amplifications of *TRAF3* and *E2F1*, but those genes do not seem to have a role in ESCC carcinogenesis [126]. Structural alterations of *TP53* and *RB1* have also been described in HNSCC [126].

### 5.2. Gene Mutations

*TP53* is the most common mutated gene in HNSCC, as well as in ESCC. Mutations of *TP53* have been described in more than 80% of HPV-negative HNSCCs and they seem to be early events in HNSCC carcinogenesis [125,126]. In HPV-positive HNSCC, *TP53* mutations are uncommon, due to p53 degradation by the viral protein E6 [128]. Similarly, the viral oncoprotein E7 is responsible of *RB1* product degradation, having a prominent role in HPV-positive HNSCC carcinogenesis [129]. Mutations of *RB1* have been described in 3% of HNSCC and seem to be early events in HNSCC development and similar data have been found in ESCC (vide supra) [125,126]. Mutations of *CDKN2A* (22% of HNSCCs), *FAT1* (23%) and *AJUBA* (6%) have been described predominantly in HPV-negative HNSCC [126]. Inactivating mutations of *NOTCH1-3* have been described in 17% of HPV-positive and 26% of HPV-negative HNSCC [125,126]. Interestingly, NOTCH family proteins seem to play a role as tumor suppressors in HNSCC, as they probably do in ESCC. Mutations of *PIK3CA* have been reported in nearly 16% of HNSCCs [129]. Finally, *MLL2*, *ZNF750*, *TGFBR2* and *FBXW7* mutations have also been described in HNSCC, but their clinical impact is unclear [126].

### 5.3. Epigenetic Alterations

Some epigenetic alterations are shared by ESCC and HNSCC. *CDKN2A* hypermethylation has been identified in HNSCC [130]. Furthermore, HNSCCs have a panel of dysregulated miRNAs which is partially similar to that of ESCC: in particular upregulation of miR-9, -21, -96, -130 and -155 and downregulation of miR-29, -31, -34, -133a, -133b, -138, -139, -143, -200, -203, -205, -218 and -375 have been reported in both malignancies [88,131,132].

## 6. Conclusions

Worldwide, ESCC still represents a deadly neoplasm. Tobacco, alcohol and PAHs consumption are some of the most frequent recognized major environmental risk factors, while congenital predisposition is well established in some syndromes. Despite studying for years of ESCC molecular characterization, no specific targeted therapies have been introduced in clinical practice. Only SOX2 inhibitors and PI3K pathway regulator drugs have been investigated without definitive and promising results. In recent years, a great number of new molecular actors playing a role in ESCC’s pathogenesis have been demonstrated, revealing ESCC as a multifaceted disease. Primary and secondary preventions still remain the first step against ESCC onset and developing non-invasive biomarkers suitable for screening purpose (e.g., circulating miRNAs) could be useful in the detection of early stage carcinomas. In cells lines, overexpression of specific miRNAs has been associated with resistance to platinum-based chemotherapy or, in a phase II study, with response to nivolumab; combined downregulation of other demonstrated increase sensitivity to paclitaxel-based chemotherapy. Future studies are warranted to study the introduction of immune checkpoints inhibitors in high molecular mutational load ESCCs and the introduction of new molecular targeted therapies, as EGFR inhibitors and mTOR pathway modulators.

## Figures and Tables

**Table 1:** Significantly mutated genes in ESCC observed in whole-genome and whole-exome studies.

Author	SMGs	Number of ESCCs
Gao et al. (2014) [32]	*TP53*, *NOTCH1*, *NFE2L2*, *MLL2*, *CDKN2A*, *RB1*, *FBXW7*, *AJUBA*, *CREEBP*, *PTCH1*	113
Song et al. (2014) [30]	*TP53*, *NOTCH1*, *NFE2L2*, *CDKN2A*, *PIK3CA*, *RB1*, *FAM135*, *ADAM29*	88
Lin et al. (2014) [31]	*TP53*, *NOTCH1*, *NFE2L2*, *MLL2*, *CDKN2A*, *ZNF750*, *PIK3CA*, *RB1*, *PTEN*, *FAT1*, *EP300*, *FAT2*, *KDM6A*	139
Zhang et al. (2015) [25]	*TP53*, *CDKN2A*, *NOTCH1*, *ZNF750*, *AJUBA*, *PIK3CA*, *FBXW7*, *FAT1*, *PTCH1*, *CREBBP*, *PTCH1*, *BAP1*	104
Sawada et al. (2016) [33]	*TP53*, *NOTCH1*, *NFE2L2*, *MLL2*, *CDKN2A*, *ZNF750*, *PIK3CA*, *FAT1*, *EP300*, *FBXW7*, *AJUBA*, *CREBBP*, *NOTCH3*, *TGFBR2*	144
Du et al. (2017) [26]	*TP53*, *NOTCH1*, *NFE2L2*, *CDKN2A*, *MLL2*, *RB1*, *ZNF750*, *PIK3CA*, *AJUBA*, *CREBBP*, *FBXW7*, *PTEN*, *FAT1*, *CUL3*, *NOTCH3*, *KDM6A*, *TGFBR2*, *DCDC1*	490
TCGA (2017) [29]	*TP53*, *NOTCH1*, *NFE2L2*, *MLL2*, *ZNF750*, *TGFBR2*	97
Li et al. (2018) [28]	*TP53*, *NOTCH1*, *NFE2L2*, *CDKN2A*, *MLL2*, *RB1*, *ZNF750*, *PIK3CA*, *AJUBA*, *CREBBP*, *EP300*, *FBXW7*, *PTEN*, *FAT1*, *CUL3*, *NAV3*, *NOTCH3*, *TENM3*, *PTCH1*, *KDM6A*, *TET2*, *TGFBR2*, *RIPK4*, *PBRM1*, *USP8*, *BAP1*	549

ESCC: esophageal squamous cell carcinoma; SMGs: significantly mutated genes; TCGA: The Cancer Genome Atlas.

**Table 2:** Principal dysregulated miRNAs in ESCC.

Onco miRNAs		Tumor Suppressive miRNAs		
miRNA	Target Gene	miRNA		Target gene
miR-21	*PTEN* *PDCD4*	miR-27a		*KRAS*
miR-130b	*PTEN*	miR-29 c		cyclin E
miR-183	*PDCD4*	miR-100		mTOR
miR-200c	*PPP2R1B*	miR-124		*STAT4*
miR-330	*PDCD4*	miR-126		*ADAM9* *IRS-1* *GOLPH3*
miR-373	*LATS2*	miR-133a/b		*FSCN1*
miR-508	*PTEN*	miR-139		*NR5A2*
miR-577	*TSGA10*	miR-143		*ERK-5*
miR-889	*DAB2IP*	miR-145		*FSCN1*
		miR-195		cdc42
		miR-203		*CDK6* DeltaNp63
		miR-375		*IGFR1*
miR-9	E-cadherin	miR-29b		*MMP-2*
miR-25	E-cadherin	miR-101 miR-126 miR-133a		*MALAT1* *ADAM9* *MMP14*
miR-92a	E-cadherin	miR-140		Slug
miR-96	*RECK*	miR-150		*ZEB1*
miR-1170	*SLIT2*	miR-200b		Kindlin-2
miR-1290	*SCAI*	miR-205		*ZEB2*
		miR-217		*MALAT1*
		miR-665		*ZEB1* *PTTG1*
miR-16	*SOX6*	miR-31		*SOX4*
miR-208	*SOX6*	miR-34a		Yin Yang-1
		miR-138		*NF-KB*
		miR-625		*SOX2*
		miR-98		*EZH2*
		miR-203		*BMI1*
		miR-214		*EZH2*
		miR-218		*EZH2*
miR-155	*TP53INP1*	miR-494		*CLPTM1 L*
miR-508	*INPP5J* *INPP4A*	miRNA-134		*FOXM1*
miR-27a/b	-			
miR-34b	-			
miR-146 b	-			
Gene involved in:	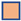	Proliferative signaling	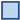	Invasion and metastasis
	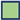	Transcriptional activator	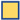	Chromatin modification
	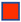	Resisting cell death	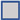	Others
